# The Italian Perinatal Surveillance System SPItOSS: insights from Confidential Enquiries

**DOI:** 10.1186/s13052-024-01811-w

**Published:** 2024-12-04

**Authors:** Michele Antonio Salvatore, Silvia Salvi, Paola D’Aloja, Patrizia Vergani, Roberto Bellù, Carlo Dani, Federico Mecacci, Maria Rosa D’Anna, Sergio Ferrazzani, Giuseppe Battagliarin, Piermichele Paolillo, Simonetta Picone, Luca Ramenghi, Giovanni Vento, Serena Donati, The Italian Perinatal Surveillance System-SPItOSS Working Group, Sara Albolino, Flavia Alessandra Rossi, Gaetano Bulfamante, Nicola Cassata, Francesca Castiglione, Giulia Dagliana, Gabriella Dardanoni, Giuseppe Ettore, Giuseppe Ferlazzo, Sebastiana Ferraro, Luigi Gagliardi, Eloisa Gitto, Paola Goretti, Giuseppe Gramaglia, Ester Grismondi, Laura Iannuzzi, Faustina Lalatta, Lucia Lo, Tommaso Mannone, Anna Maria Marconi, Emiliano Maresi, Massimo Micaglio, Alessandra Moretto, Fabio Mosca, Giuseppe Murolo, Elisabetta Pelo, Maria Piccione, Simone Pratesi, Emanuele Scarpuzza, Caterina Serena, Filiberto Maria Severi, Francesca Strigini, Nicola Strobelt, Paolo Emilio Tagliabue, Rosaria Taverna, Barbara Tomasini, Marcello Vitaliti, Fabio Voller, Giovanni Bartoloni

**Affiliations:** 1 National Centre for Disease Prevention and Health Promotion, Istituto Superiore di Sanità - Italian National Institute of Health, Viale Regina Elena 299, Rome, 00161 Italy; 2 UOC Ostetricia e Patologia Ostetrica, Dipartimento di Scienze della Salute della Donna e del Bambino e di Sanità Pubblica, Fondazione Policlinico Universitario A. Gemelli, IRCCS, Largo A. Gemelli 8, Rome, 00168 Italy; 3 Fondazione Monza e Brianza per il Bambino e la sua Mamma, Ospedale San Gerardo, Università Degli Studi Di Milano-Bicocca, Milan, Italy; 4 Ospedale Manzoni, Lecco, Italy; 5 Division of Neonatology, Azienda Ospedaliero-Universitaria Careggi, Florence, Italy; 6 Department for Women and Children Health, Azienda Ospedaliero-Universitaria Careggi, Florence, Italy; 7 Ospedale Buccheri La Ferla, Palermo, Italy; 8 Comitato “Percorso Nascita della Regione Emilia Romagna”, Bologna, Italy; 9 Neonatology and Neonatal Intensive Care Unit, Policlinico Casilino, Rome, Italy; 10 Neonatal Pathology Unit, Istituto Giannina Gaslini, Genoa, Italy; 11 Neonatology Unit, Fondazione Policlinico Universitario A. Gemelli, IRCCS, Dipartimento Scienze della Salute della Donna, del Bambino e di Sanità Pubblica, Rome, Italy

**Keywords:** Perinatal death, Neonatal death, Maternal surveillance, Neonatal surveillance, Confidential enquiry, Perinatal mortality review, Active surveillance, Perinatal care

## Abstract

**Background:**

An effective strategy to reduce perinatal mortality requires an active surveillance system. This includes monitoring cases, organizing multidisciplinary local audits, conducting Confidential Enquiries, identifying avoidable factors, and facilitating changes in the healthcare system. In 2017, the Italian Obstetric Surveillance System launched the SPItOSS pilot Perinatal Surveillance System. The aim of this paper is to describe the results of the SPItOSS Confidential Enquiries on perinatal deaths focusing on the emergent critical aspects in obstetric and neonatal care, as well as on the healthcare facilities organization.

**Methods:**

SPItOSS, a population-based surveillance system, collected and analysed incident perinatal deaths from July 2017 to June 2019 in three Regions encompassing 32.3% of Italian births. Cases were defined according to WHO definition as fetuses born dead ≥ 28 weeks of gestation and live newborn died within 7 days from birth. The International Statistical Classification of Diseases and related Health Problem-Perinatal Mortality was adopted for coding causes of death and contributing maternal and placenta-related conditions. Confidential Enquiries, prioritized according to perinatal deaths preventability, were conducted by expert committees at Regional and National level.

**Results:**

A total of 830 incident perinatal deaths were notified, with 58.3% classified as antepartum, 4.3% as intrapartum, and 37.3% as neonatal deaths. According to the SPItOSS protocol, Confidential Enquiries evaluated only the most preventable deaths, including 19 intrapartum and 70 neonatal deaths. Of these, 43.8% were assessed as unavoidable with appropriate care; 29.2% as unavoidable with improvable care, and 15.7% as avoidable due to inappropriate care. Most intrapartum deaths were attributed to intrauterine hypoxia, while neonatal deaths recognized a multifactorial aetiology. Different aspects of inappropriate care were highlighted, such as failure to recognise maternal or fetal problems before labour, delayed or inappropriate neonatal resuscitation, and poor or suboptimal neonatal monitoring.

**Conclusions:**

The SPItOSS Confidential Enquires provided insights for improving maternity and perinatal services. By targeting key areas of obstetric and neonatal care, the surveillance can generate recommendations and actions to prevent avoidable perinatal deaths.

## Background

Over the last decade, clinicians, researchers, public health advocates, and parents’ groups have raised the profile of perinatal deaths as a public health problem. The World Health Organization (WHO) provided guidance and frameworks for developing perinatal mortality surveillance systems that capture every death and gather data on underlying causes and preventable factors [1, 2]. Consequently, the reduction of perinatal and child mortality has been introduced as one of the eight Millennium Development Goals and as a key objective in the 17 Sustainable Development Goals 2016–2030 [3].

The understanding of perinatal mortality and the implementation of preventive measures have been globally hindered by the lack of universally accepted definitions. To address this issue, the WHO recommended a unified definition of perinatal mortality in 2006 [4]. Likewise, regarding identifying the causes of perinatal mortality, the International Statistical Classification of Diseases and related Health Problem-Perinatal Mortality (ICD-PM) classification system, based on the 10th revision of the International Statistical Classification of Disease, gained global acceptance and widespread application [5]. This system provides a user-friendly approach to categorize the timing (antepartum, intrapartum, neonatal) and the causes of perinatal deaths linked to underlying maternal conditions [5–7].

An effective strategy to reduce perinatal mortality must prioritize the key component of an active surveillance system. This includes monitoring the incidence of cases, conducting local audits and Confidential Enquiries; identifying avoidable factors, and, when needed, enabling changes at every level of the health and social care system [4; 8]. At global level, various countries and Regions have established their own perinatal mortality surveillance systems or are in the process of establishing them to track and analyse data related to perinatal deaths [8], however Italy has not yet established a National surveillance. In Europe, the MBRRACE-UK (Mothers and Babies: Reducing Risk through Audits and Confidential Enquiries across the UK) Mortality Surveillance is a well-established maternal and perinatal surveillance system based on incident reporting and Confidential Enquiries [9]. Inspired by the MBRRACE UK [10], in 2017, the Italian Obstetric Surveillance System (ItOSS) [11, 12] launched the SPItOSS Perinatal Surveillance System pilot project in three Italian regions [13, 14].

The aim of this paper is to describe the findings from the SPItOSS Confidential Enquiries, focusing on critical aspects in obstetric and neonatal care as well as in healthcare facility organization, and to identify aspects that could improve care and reduce avoidable perinatal deaths.

## Material and methods

The SPItOSS population-based pilot project collected and analysed incident cases of perinatal deaths occurred from July 2017 to June 2019 in three Italian Regions representing the northern, central, and southern parts of the country, namely Lombardy, Tuscany, and Sicily. In 2015, these Regions accounted for 32.3% of National births. All maternity, neonatal and intensive care units (NICU) of these Regions joined the project.

According to the WHO definition of perinatal death [4], SPItOSS recorded incident cases of stillbirths classified as babies born with no signs of life ≥ 28 weeks of gestation and neonatal deaths within 7 days from delivery [13].

The ICD-PM was adopted to code and categorize these deaths based on timing, causes, and related maternal and placenta-related conditions [5].

For each perinatal death, the hospital’s reference clinicians and risk manager organized a multi-professional audit involving the entire staff associated with the case, to evaluate the underlying causes of death, using the Significant Event Audit (SEA) methodology [15].

Panels of experts, comprising obstetricians, neonatologists, midwives, geneticists, pathologists, and risk managers, reviewed a chosen subset of deaths through both Regional and National Confidential Enquiries. These Confidential Enquiries assessed the quality of care provided in individual cases against evidence-based guidelines or accepted best practices.

Given the number of events and available resources, the SPItOSS selection protocol defined the prioritization of cases according to the estimated preventability of each death and established that 100 was the maximum number of Confidential Enquiries feasible during the study period [13]. Intrapartum deaths were given the highest priority, followed by neonatal deaths occurring from 28 gestational weeks to the 7th day of life, firstly those related to intrapartum fetal distress. Antepartum deaths were excluded due to limited resources for their examination [13]. Additionally, fetal anomalies incompatible with life, as well as congenital malformations with no life expectancy were excluded from Confidential Enquiries.

During the enquiry, an anonymous paper form was filled in by the Confidential Enquiries Regional Committee (CERC) experts and subsequently transferred, along with the complete clinical documentation, to the National Operational Unit (NOU) for the project coordination, led by the Istituto Superiore di Sanità – Italian National Institute of Health (INIH), twice a year. The perinatal deaths were successively re-evaluated by the Confidential Enquiries National Committee (CENC). During the central Confidential Enquiries, an anonymous paper form was filled in by the CENC experts, appointed by INIH. Annually, the NOU convened a national conference, involving experts from the CERC and the CENC to review and jointly evaluate cases with discrepant assessments between Regional and National level. Death preventability was evaluated according to predefined grading criteria [13] and adapted from the CESDI (Confidential Enquiry into Stillbirths and Deaths in Infancy) grading [16]: Grade 1. Inappropriate care with avoidable outcome; Grade 2. Improvable care with unavoidable outcome; Grade 3. Appropriate care with unavoidable outcome; Unaccountable.

### Statistical analysis

Frequency distributions and prevalence rates were used to analyse maternal characteristics, causes of death, including contributing maternal and placental-related conditions, and assess preventability. This analysis was conducted in alignment with the described grading system for intrapartum and neonatal deaths revised through Confidential Enquiries. Statistical analyses were performed using STATA/MP version 14.2

## Results

**Fig. 1:**
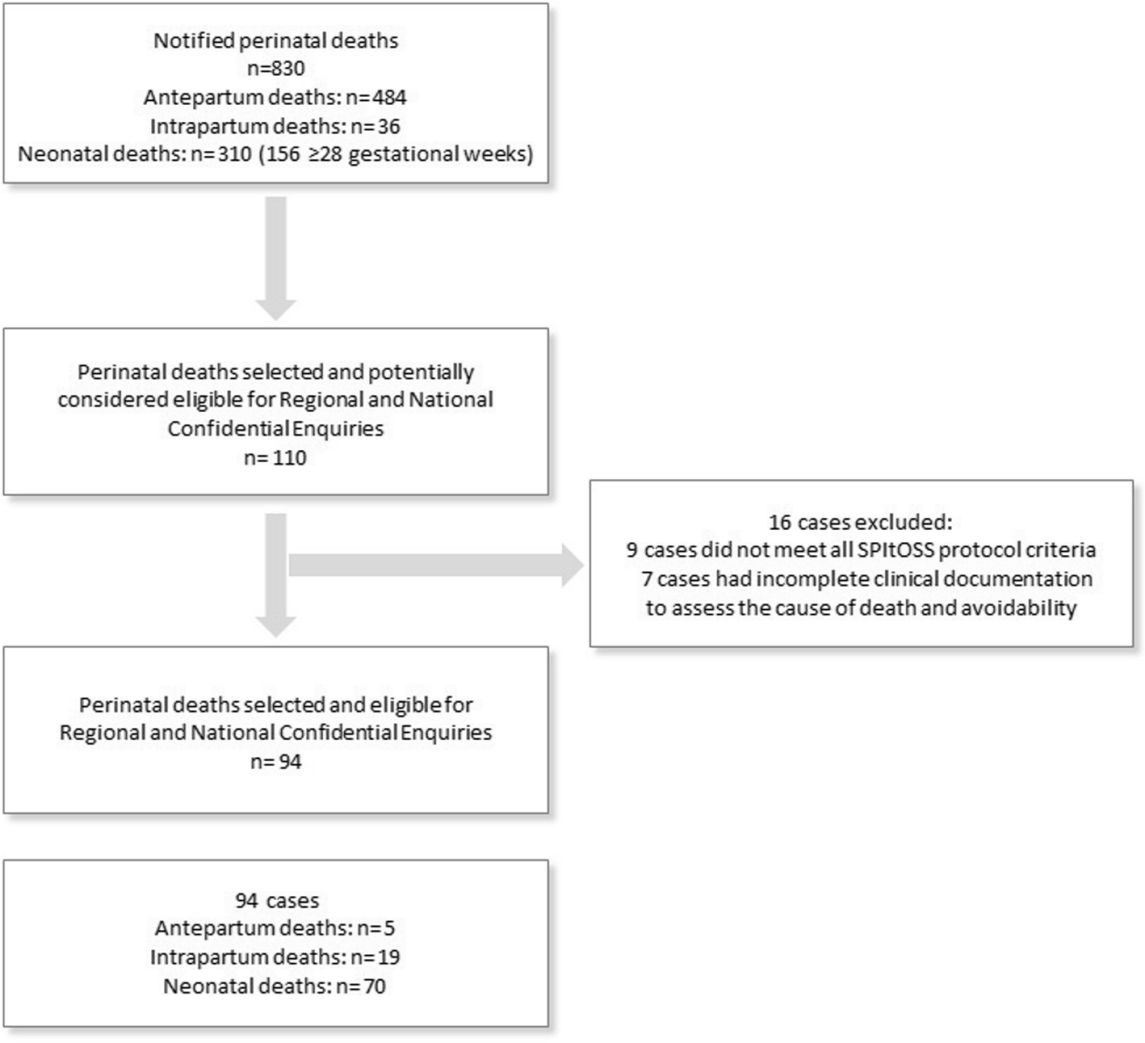
Perinatal deaths revised through Confidential Enquiries

During the two-year pilot surveillance period, 830 incident perinatal deaths were notified across the three participating Regions. Among these cases, 58.3% were classified as antepartum deaths (*n* = 484), 4.3% as intrapartum deaths (*n* = 36) and 37.3% (*n* = 310) as neonatal deaths (Fig. 1).

According to the SPItOSS selection protocol criteria for which intrapartum deaths received the highest priority followed by neonatal deaths, 110 perinatal deaths (13.3%) were potentially considered eligible for Regional and National Confidential Enquiries. After excluding 16 inapplicable cases, the remaining 94 were assessed through the entire Confidential Enquiries process (Fig. 1).

As previously described in detail [13], this process involved 15 meetings per year of the Regional CERC, 11 meetings of the National CENC, and 2 collegial meetings to reach consensus on 23 (24.5%) cases emerged as discrepant between the Regional and National assessment.

Of the 94 perinatal deaths evaluated by Regional and National Confidential Enquiries, this analysis focused on 89 cases: 19 intrapartum deaths and 70 neonatal deaths. The remaining five antepartum deaths were excluded because erroneously included in the pool eligible for Confidential Enquiries due to a timing error in the recorded dates of their deaths, which was discovered only during their review (Fig. 1).

Notifications of the 89 cases originated from 42 maternity units comprising 29 hubs (equipped with NICU and with > 1000 deliveries per year), 12 spokes (without NICU and with < 1000 deliveries per year) and one facility for which no information were available.

Among the 89 cases, 31.4% of the women were aged 35 or older, 62.9% were multiparous, and 33.7% were of foreign citizenship. The percentage of multiple pregnancies was 4.5%.

Causes of death and maternal and placental related conditions for intrapartum and neonatal deaths were respectively illustrated in Tables 1 and 2. The majority of intrapartum deaths (12/19) were attributed to intrauterine hypoxia, with intrapartum infections accounting for 4 out of 19 cases, as detailed in Table 1. On the contrary, neonatal deaths were found to have a multifactorial aetiology, with three leading causes being respiratory and cardiovascular disorders (20/70), complications of intrapartum events (15/70), and infections (13/70), as shown in Table 2.

**Table 1:** Distribution of intrapartum deaths revised through Confidential Enquiries by cause, maternal and placental related conditions

Cause of death *and maternal and placental related conditions*	n	%
Intrapartum acute events	12	63.2
Intrauterine hypoxia	12/12	
*Maternal and placental related conditions:*		
*other forms of placental separation and haemorrhage*	7/12	
*prolapsed cord, other compression of umbilical cord*	1/12	
*other labor and delivery complications*	1/12	
*preterm rupture of membranes*	1/12	
*maternal diabetes, including gestational diabetes*	1/12	
*non-maternal associated condition*	1/12	
Infections	4	21.1
Other perinatal infections	2/4	
Neonatal bacterial sepsis	1/4	
Not specified	1/4	
*Maternal and placental related conditions:*		
*chorioamnionitis*	4/4	
Other intrapartum conditions	1	5.3
Foetal blood loss	1/1	
*Maternal and placental related conditions:*		
*other membrane complications*	1/1	
Unspecified other intrapartum deaths	2	10.5
Unspecified other intrapartum deaths	2/2	
*Maternal and placental related conditions:*		
* polyhydramnios/oligohydramnios*	1/2	
* other pregnancy complications*	1/2	
Total	19	100.0

**Table 2:** Distribution of neonatal deaths revised through Confidential Enquiries by cause, maternal and placental related conditions

Cause of death *and maternal and placental related conditions*	n	%
Respiratory and cardiovascular disorders	20	28.6
Neonatal respiratory distress	8/20	
Meconium aspiration syndrome	5/20	
Other perinatal respiratory disturbances	2/20	
Perinatal cardiovascular disorders	2/20	
Unspecified	3/20	
*Maternal and placental related conditions:*		
*non-maternal associated condition*	6/20	
*chorioamnionitis*	4/20	
*nutritional disorders*	2/20	
*other forms of placental separation and haemorrhage*	1/20	
*prolapsed cord, other compression of umbilical cord*	1/20	
*preterm labor and delivery*	1/20	
*pre-eclampsia/eclampsia*	1/20	
*maternal diabetes, including gestational diabetes*	1/20	
*not specificied maternal conditions*	1/20	
*not defined maternal conditions*	2/20	
Complications of intrapartum events	15	21.4
Birth asphyxia	10/15	
Intrauterine hypoxia	5/15	
*Maternal and placental related conditions:*		
*other forms of placental separation and haemorrhage*	4/15	
*non-maternal associated condition*	3/15	
*placental dysfunction*	1/15	
*prolapsed cord, other compression of umbilical cord*	1/15	
*chorioamnionitis*	1/15	
*other membrane complications*	1/15	
*other pregnancy complications*	1/15	
*operative vaginal delivery*	1/15	
*maternal diabetes including gestational diabetes*	1/15	
*not specificied maternal conditions*	1/15	
Infections	13	18.6
Neonatal bacterial sepsis	10/13	
Congenital pneumonia	2/13	
Other perinatal infections	1/13	
*Maternal and placental related conditions:*		
*chorioamnionitis*	7/13	
*non-maternal associated condition*	3/13	
*preterm rupture of membranes*	1/13	
*infectious and parasitic diseases*	1/13	
*not specificied maternal conditions*	1/13	
Congenital malformations, deformations and chromosomal abnormalities	9	12.9
*Maternal and placental related conditions:*		
*non-maternal associated condition*	6/9	
*polyhydramnios/oligohydramnios*	2/9	
*prolapsed cord, other compression of umbilical cord*	1/9	
Disorders related to length of gestation and low birthweight	5	7.1
*Maternal and placental related conditions:*		
*preterm labor and delivery*	1/5	
*other forms of placental separation and haemorrhage*	1/5	
*chorioamnionitis*	1/5	
*polyhydramnios/oligohydramnios*	1/5	
*pre-eclampsia/eclampsia*	1/5	
Disorders related to fetal growth	1	1.4
*Maternal and placental related conditions:*		
*gestational hypertension*	1/1	
Other neonatal conditions	4	5.7
*Maternal and placental related conditions:*		
*preterm labor and delivery*	2/4	
*pre-eclampsia/eclampsia*	1/4	
*non-maternal associated condition*	1/4	
Other conditions	2	2.9
*Maternal and placental related conditions:*		
*non-maternal associated condition*	2/2	
Not identifiable causes	1	1.4
Total	70	100.0

Placental abruption and haemorrhage were the main maternal conditions associated with acute intrapartum events, whereas a diagnosis of chorioamnionitis was overall present in intrapartum deaths related to infection (Table 1). On the contrary, less than one third (6/20) of the neonatal deaths attributed to respiratory and cardiovascular disorders had no maternal associated condition (Table 2).

The Regional and National Confidential Enquiries assessed as unavoidable with appropriate care 39 perinatal deaths (43.8%); 26 (29.2%) were judged as unavoidable with improvable care and 14 (15.7%) as avoidable with inappropriate care. Cases assessed as unaccountable were 10 (11.2%). Significant Regional variation was found in the proportion of avoidable deaths, ranging from zero in Tuscany (Centre of Italy) to 12.2% (n = 5/41) in Lombardy (Northern Italy), and 37.5% (n = 9/24) in Sicily (Southern Italy). Intrapartum deaths represented 22.2% (2/9) of the avoidable deaths in Sicily and 40.0% (2/5) in Lombardy.

Table 3 summarizes the nature of inappropriate care episodes observed in perinatal deaths deemed avoidable. The most common issue was the failure to recognise maternal or fetal problems upon admission or before labour. This was followed by inadequate actions post- labour, such as delayed or inappropriate neonatal resuscitation and poor or inappropriate neonatal monitoring*.* In ten out of 15 cases, these issues likely had a significant impact on neonatal outcomes. Additionally, inappropriate conduct during labour induction was frequently noted, as were various organizational failures, including delays in scheduling Caesarean section and failure to refer patients to higher-level care when necessary.

**Table 3:** Failures or delays identified in 15 avoidable perinatal deaths, as evaluated through Confidential Enquiries^a^

Nature of the detected inappropriate care	*n*	%
Failure to recognise a problem at admission/before labour	20	41.7
*Delayed/inappropriate diagnosis*	5	
*Poor/inappropriate surveillance*	7	
*Abnormal CTG*	5	
*Delays in communication among health professionals*	3	
Failure to recognise a problem during labour	1	2.1
*Abnormal CTG or meconium*	1	
Failure to act appropriately during labour	7	14.6
*No CTG performed despite indications*	1	
*Poor quality CTG*	2	
*Uterine stimulation despite adverse effects on fetal heart rate*	3	
*Delays in communication among health professionals*	1	
Failure to act appropriately after labour	10	20.8
*Delayed/inappropriate neonatal resuscitation*	4	
*Poor/inappropriate neonatal surveillance*	6	
Failure to act appropriately during Cesarean Section	3	6.3
*Incorrect type of anestesia*	2	
*Delay in fetal extraction/improper extraction*	1	
Failure in healthcare organization	7	14.6
*Inadequate hospital level of care*	1	
*Delay in organizing Cesarean section (operating room unavailability)*	2	
*Delay in organizing Cesarean section (difficulty in calling the second surgeon)*	1	
*Delay in organizing Cesarean section (difficulty in calling the anesthetist)*	1	
*Delay in organizing Cesarean section (unavailability of neonatologist)*	2	
Total instances of failures or delays	48	100.0

^a^A single case can involve multiple instances of failures or delays

**Fig. 2:**
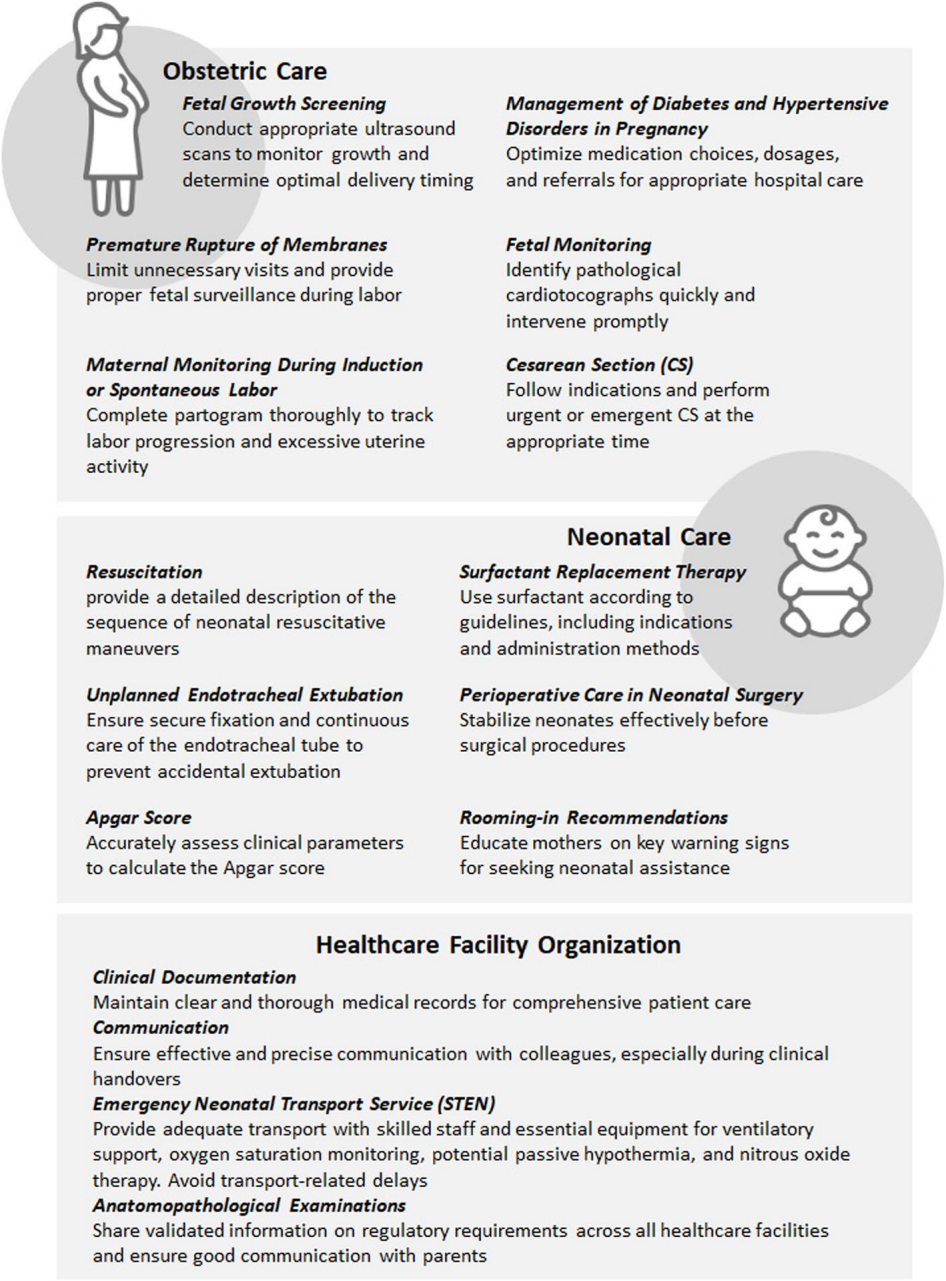
Clinical practice recommendations from SPItOSS Confidential Enquiries

Figure 2 summarizes the different clinical practice recommendations arising from the Confidential Enquiries of all 89 analysed cases, covering antenatal and intrapartum care, neonatal resuscitation, and healthcare organization.

## Discussion

### Principal findings

Active surveillance through Confidential Enquiries enabled a comprehensive evaluation of perinatal deaths, assessing both the causes of death and the quality of care provided. Assessments indicated that improvements in care could have impacted the child's outcomes in 45% of the cases. Among avoidable cases, the most common issues were failure to recognise maternal or fetal problems upon admission or before, as well as inadequate actions after labour in terms of delayed or inappropriate neonatal resuscitation and poor or inappropriate neonatal surveillance. Figure 2 presents the overall findings, describing different aspects in pregnancy and intrapartum care, neonatal resuscitation, and general healthcare assistance.

### Strengths and limitations

This pilot project enabled the first systematic review of perinatal deaths through Confidential Enquiries in three Italian Regions. Its strength stems from its population-based approach and its adherence to the WHO-recommended definition and classification of perinatal death causes [4, 5]. The multidisciplinary composition of the enquiry panels ensured a comprehensive evaluation of cases based on evidence-based guidelines or accepted best practices.

A potential limitation is the exclusion of births below 28 weeks affecting a proportion of potentially viable foetuses, especially in high-income countries. However, the adoption of the WHO definition facilitated comparisons with other countries, given the significant variability in the distinction between abortion and birth among different nations. Moreover, the sub-National design represents a limitation, although the three participating regions are geographically distributed across the North, Central, and South of the country and involve one third of national births.

It is essential to acknowledge that no definitive conclusions can be drawn about the prevalence of similar care-related issues in births where the baby did not die. Nevertheless, the identified improvements in care and health care organization have the potential to impact all women and newborns receiving care.

### Interpretation

According to the last Euro-Peristat Report [17], Italy's perinatal outcomes excel beyond the European average. In 2019, the Italian stillbirth rate (> 24 weeks of gestation) was 2.7 per 1000 births, as opposed to the European median of 3.2. Additionally, the neonatal mortality rate (> 22 weeks) stood at 1.7 deaths per 1000 live births, against the European median of 2.1 [17]. In 2017, the Italian Obstetric Surveillance System (ItOSS) [11, 12] launched the SPItOSS pilot project, drawing inspiration from the MBRRACE-UK model for conducting Confidential Enquiries into perinatal deaths [10].

Although intrapartum deaths make up just 4.3% and 2.2% of all deaths reported to SPItOSS and in the latest MBBRACE-UK report [18], respectively, reviewing them is crucial for helping parents understand what happened and enabling maternity units to learn and improve. In our cohort, intrauterine hypoxia was the principal cause of intrapartum deaths related in 63% of cases to placental abruption. Infections explain another 21% of cases always associated to maternal chorioamnionitis.

The most common causes of neonatal death detected by the MBRRACE-UK were congenital anomalies, extreme prematurity, neurological, cardio-respiratory and infectious diseases [18]. Among the 70 SPItOSS neonatal cases, respiratory and cardiovascular disorders (28.6%), and complications of intrapartum events (21.4%), infections (18.6%) and congenital malformations/chromosomal anomalies (12.9%) were the most acknowledged causes of death. The latter exhibits a lower occurrence than expected, owing to the a priori exclusion from Confidential Enquiries of fetal anomalies or congenital malformation leading to no life expectancy, in accordance with the SPItOSS protocol described in the methods section. Discrepancies in other causes seem to stem primarily from distinct classification systems; MBRRACE- UK employs the Cause of Death & Associated Conditions (CODAC), while SPItOSS utilizes the ICD- PM.

Expert panel recommendations for all 89 cases are synthetized in Fig. 2. Clinical key findings from the enquiries in antenatal, intrapartum and neonatal care reveal common themes often recurring in other perinatal mortality active surveillances [19]. Notably, a significant gap was identified in recognizing and managing gestational diabetes, which increases the risk of adverse neonatal outcomes [20, 21]. Similarly, critical issues identified regarding hypertensive disorders of pregnancy (HDP) include delayed diagnosis, insufficient maternal risk assessment, and inadequate healthcare service levels [22]. Given that premature births achieve better outcomes in maternity units with on-site neonatal intensive care [23], it is concerning that 29% of the maternity units reporting cases reviewed through Confidential Enquiries were classified as spoke units. Interestingly, in Tuscany (Central Italy), where there were no spoke maternity units involved, no avoidable deaths occurred.

In cases of congenital malformations, the lack of accurate prenatal diagnosis hindered the improvement of neonatal outcomes. This absence prevented the alignment of capacity with demand and the optimization of perinatal and neonatal care [24, 25]. Detecting fetal growth restriction proved challenging due to infrequent ultrasound scans, inadequate fetal growth or Doppler assessment, and insufficient monitoring or referral to tertiary fetal medicine centres when needed [26–28]. Poor management of labour induction was a common factor in avoidable deaths, emphasizing the need for proper monitoring of maternal health, uterine contractions, and fetal heartbeat. Strict adherence to evidence-based guidelines is paramount to mitigate these risks [16, 21, 22].

Concerning neonatal care, key areas for improvement include proper indication and administration of surfactant [29]. Respiratory failure due to surfactant deficiency is a major cause of respiratory failure in preterm infants and contributes to acute respiratory morbidity among late-preterm and term neonates with conditions such as meconium aspiration syndrome, pneumonia or sepsis, and pulmonary haemorrhage [29]. Addressing these issues can greatly enhance neonatal outcomes and reduce risks. Unplanned endotracheal extubation causing hypoxia, bradycardia, and potential airway trauma requiring urgent re-intubation, was also flagged [30]. Additionally, inadequate organization of the neonatal transfer service pointed the gaps in following the protocol for managing neonatal emergencies, consistent with other author’s finding on intrapartum deaths [31].Confidential enquiries have also shed light on critical issues in healthcare organization, including staffing, space, facility infrastructure and equipment problems often linked to high activity levels. Incomplete clinical records and poor communication among clinicians, particularly during handovers, were also noted [32]. Addressing these challenges is essential for optimizing patient care and reducing risks within healthcare settings.

In line with the findings of SPItOSS, Italy shows significant and persistent disparities in neonatal and infant mortality rates between the North and the South. A recent publication indicates that from 2016 to 2020, infants born in the South experienced an infant mortality rate approximately 70% higher than those in the North, primarily due to elevated rates of neonatal respiratory distress and prematurity [33, 34]. Moreover, disparities have also been observed in neonatal and infant mortality rates between immigrant and Italian residents. In our data, nearly 34% of the mothers were foreign, compared to 21% in the general obstetric population [35].

The SPItOSS pilot project led to recommendations for clinical practice aimed at healthcare organizations and professionals. A report on the pilot surveillance was published [36], a National Congress shared the project's results, and recommendations were published on the ItOSS website, with a dedicated section for the SPItOSS project [37]. Initiatives to disseminate recommendations and raise awareness of perinatal mortality risk factors were carried out, following the experience of other countries with perinatal surveillance systems [19]. Additionally, the expert panel suggested strengthening healthcare services, particularly in the Southern Regions where previous data had already highlighted the critical issues identified by SPItOSS [38], and implementing quality initiatives within maternity and neonatal units. Following the conclusion of the pilot project, the Lombardy region, which recorded 67.000 births in 2022, opted to continue the surveillance initiative. This ongoing effort is providing valuable organizational and managerial insights, paving the way for potential expansion of surveillance efforts nationwide.

## Conclusions

The SPItOSS pilot project has proven its efficacy in identifying causes of perinatal deaths and highlighting critical issues in obstetric and neonatal care, as well as in healthcare facility organization. The Confidential Enquiries outcomes raised awareness and prompted actions to prevent avoidable perinatal deaths in participating Regions, where there is strong interest in maintaining this surveillance, considered vital for public health. Following the assessment of the SPItOSS pilot project's effectiveness and sustainability, the Italian National Institute of Health officially recommended that the Ministry of Health extend the surveillance nationwide, following a model similar to the ItOSS maternal mortality surveillance system [12].

## Data Availability

The datasets generated and analysed during the current study are not publicly available due to privacy restrictions but are available from the corresponding author on reasonable request.
